# Supplementary material for: Stable Aqueous Colloidal Solutions of Nd3+: LaF3 Nanoparticles, Promising for Luminescent Bioimaging in the Near-Infrared Spectral Range
Source: Nanomaterials (Basel). 2021 Oct 26;11(11):2847. doi: 10.3390/nano11112847 (PMC8625732; doi:10.3390/nano11112847)
Supplement: Supplementary file 1 [file nanomaterials-11-02847-s001.zip › nanomaterials-1428008-supplementary.pdf]

Supplementary Materials:

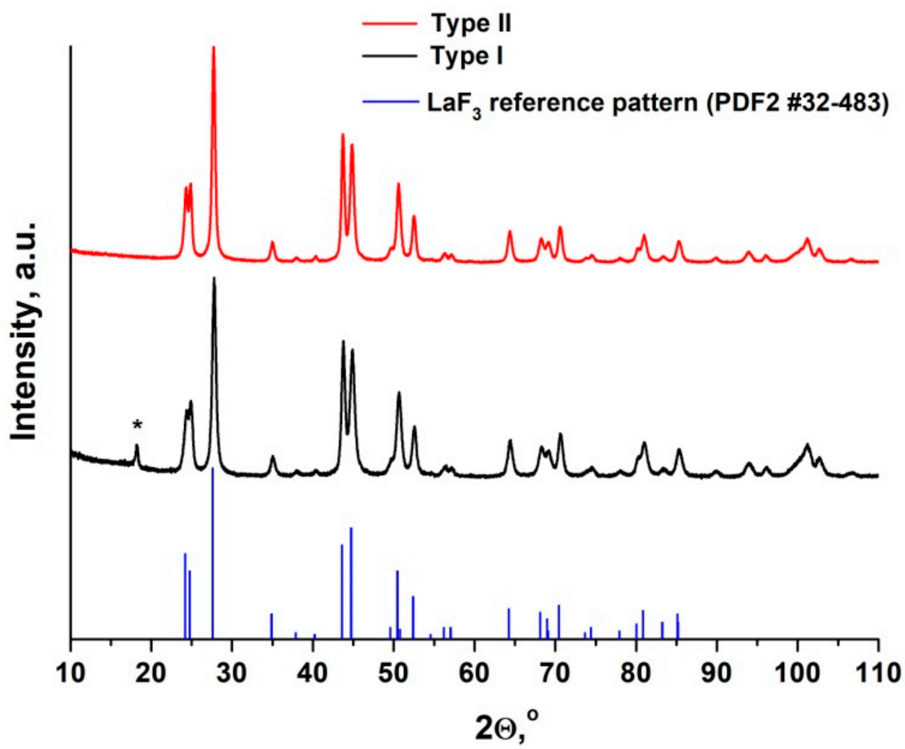

Figure S1. XRD patterns of synthesized LaF_3 NPs in comparison with the reference pattern from the ICDD database (#78-1864). (* is a reflection from the Teflon (polytetrafluorethylene) support, on which colloidal NPs were dried prior to XRD measurements).

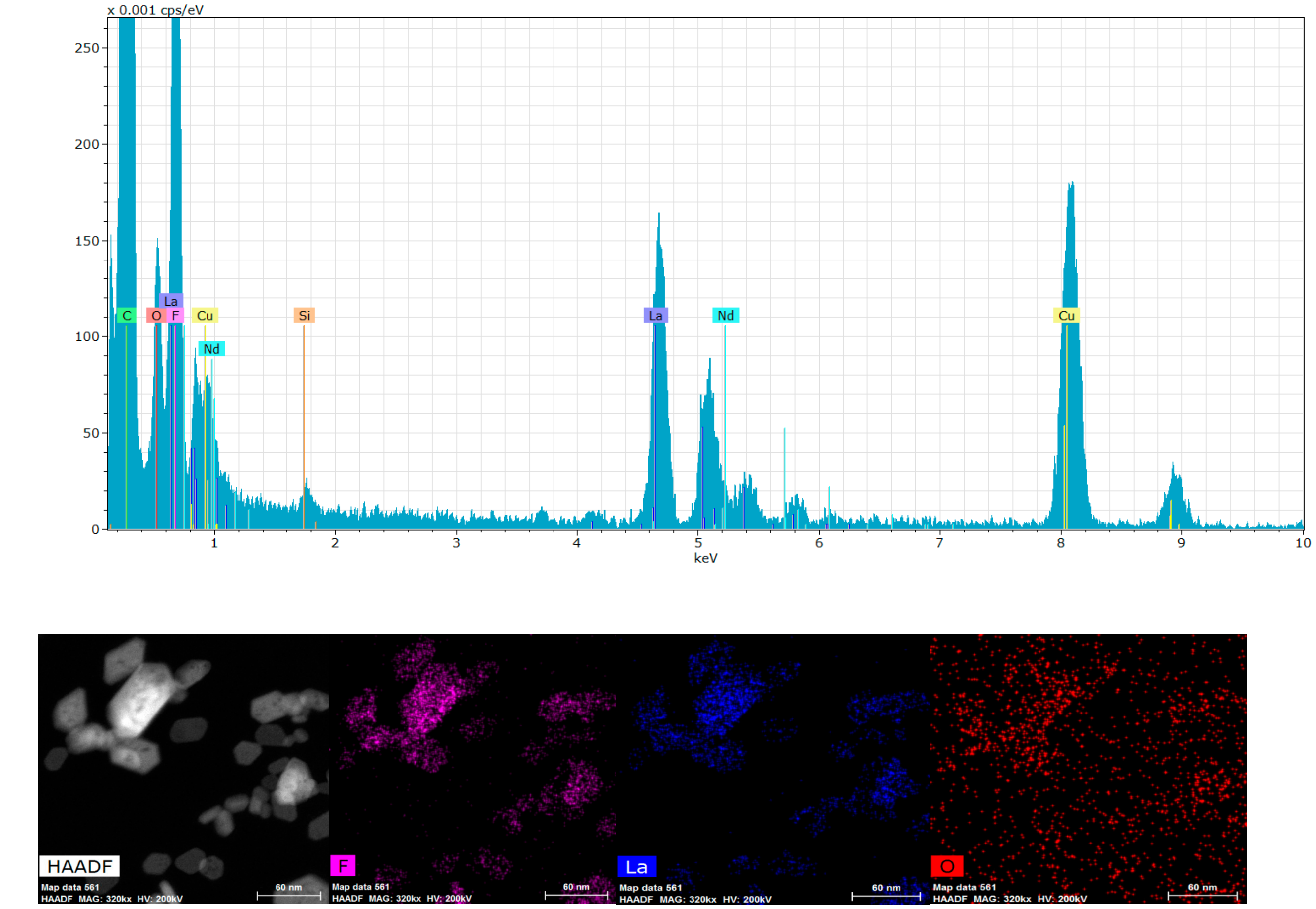

Figure S2. a. EDX spectrum (top) and microphotographs in the HAADF mode of the distribution of elements F, La, and O (oxygen) in 4 at% Nd^{3+}: LaF_3 NPs of Sample type I.

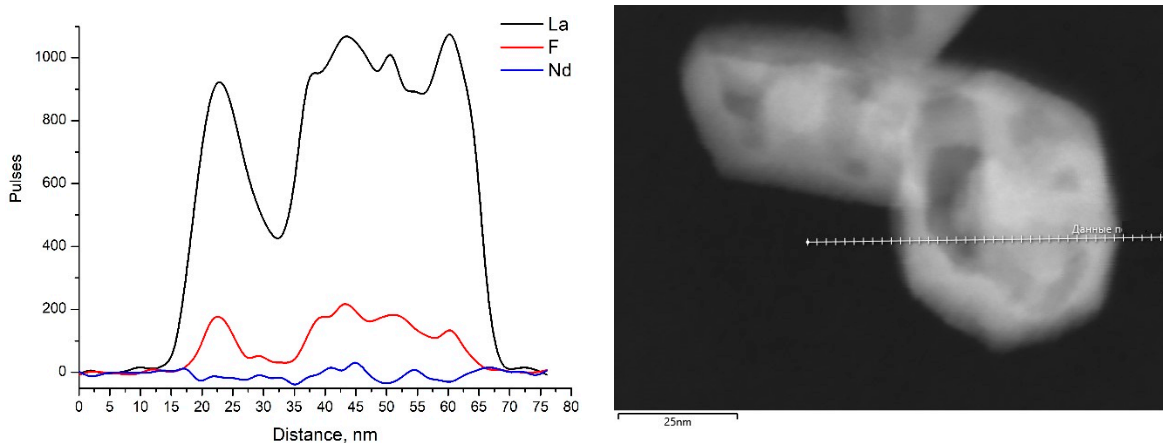

Figure S2. b. STEM image (left) and EDX distribution profile of elements (right) along the scanning line of a nanoparticle of the 1 at% Nd^{3+}: LaF_3 sample type II; the ratio of the concentrations of La (black curve) and F (red curve) atoms along the scanning line remains constant; Nd atoms are determined at the trace level (blue curve) due to its low concentration (1 at%) in this sample.

Table S1. Parameters of Nd^{3+}: LaF_3 NPs: unit cell, coherent scattering region (CSR) of X-rays by NPs, average nanoparticle sizes according to TEM results.

**Sample**	***a*, Å**	***c*, Å**	**CSD (mean size of scattering domain of NPs ($\langle D \rangle_v$), nm**	**Sizes of NPs in accordance with TEM, nm**	
				Fine fraction	Coarse fraction
0.01 *at.*% Nd^{3+}: LaF_3 (Sample type I)	7.172(1)	7.375(1)	**19 ± 1**	$D_F = 16 \pm 4$<br>$d_F = 12 \pm 3$<br>**$\langle D_{F\ sphere} \rangle = 13 \pm 4$**	$D_C = 23 \pm 6$<br>$d_C = 16 \pm 5$<br>**$\langle D_{C\ sphere} \rangle = 18 \pm 6$**
				Volume ratio (Figure 3 a)	
				$D_F:D_C = 0.53:0.47$	$d_F:d_C = 0.57:0.43$
				$(D_F+d_F)/ 2 = (0.53+0.57)/2 = 0.55$<br>$(D_C+d_C) = (0.47+0.43)/2 = 0.45$<br>$\langle D_{sphere} \rangle = 0.55 \langle D_{F\ sphere} \rangle + 0.45 \langle D_{C\ sphere} \rangle$<br>**$\langle D_{sphere} \rangle = 15.4 \pm 6$**	
2 *at*% Nd^{3+}: LaF_3 (Sample type II)	7.177(1)	7.377(1)	**24 ± 1**	$D_F = 21 \pm 4$<br>$d_F = 16 \pm 4$<br>**$\langle D_{F\ sphere} \rangle = 18 \pm 4$**	$D_C = 30 \pm 7$<br>$d_C = 23 \pm 6$<br>**$\langle D_{C\ sphere} \rangle = 25 \pm 7$**
				Volume ratio (Figure 3 b)	
				$D_F:D_C = 0.49:0.51$	$d_F:d_C = 0.64:0.36$
				$(D_F+d_F)/2 = (0.49+0.64)/2 \approx 0.57$,<br>$(D_C+d_C)/2 = (0.51+0.36)/2 \approx 0.43$<br>$\langle D_{sphere} \rangle = 0.57 \langle D_{F\ sphere} \rangle + 0.43 \langle D_{C\ sphere} \rangle$<br>**$\langle D_{sphere} \rangle = 21 \pm 7$**	

*Indices F and C correspond to the characteristic sizes for the parameters, respectively, of fine and coarse fractions of both samples of NPs; D_F, d_F and D_C, d_C are small and large diameters of model ellipses, which approximate TEM images of projections of NPs of fine and coarse fractions; $\langle D_{N\ sphere} \rangle$ are average diameters of the sphere, calculated from the condition of equality of the volume of a sphere with such a diameter $\langle D_{N\ sphere} \rangle$ and model ellipsoids obtained by rotating an ellipse with diameters D_N and d_N relative to a large diameter D_N; $\langle D_{sphere} \rangle$ values are calculated taking into account the average specific contribution of both fractions of NPs, each of which is characterized by an average diameter $\langle D_{N\ sphere} \rangle$.
